# Influence of the timing of bronchoscopic alveolar lavage on children with adenovirus pneumonia: a comparative study

**DOI:** 10.1186/s12890-021-01708-y

**Published:** 2021-11-11

**Authors:** Xue-hua Xu, Hui-feng Fan, Ting-Ting Shi, Di-Yuan Yang, Li Huang, Wen-hui Jiang, Gen Lu

**Affiliations:** 1 Department of Respiratory, Guangzhou Women and Children’s Medical Center, Guangzhou Medical University, Guangzhou, 510120 Guangdong China; 2 Pediatric Intensive Care Unit, Guangzhou Women and Children’s Medical Center, Guangzhou Medical University, Guangzhou, China

**Keywords:** Adenovirus pneumonia, Bronchoscopic alveolar lavage, Timing, Children, Comparative study

## Abstract

**Background:**

Adenovirus pneumonia is prone to severe clinical and imaging manifestations in children. Bronchoscopic alveolar lavage (BAL) is an important adjunctive therapy for patients with severe imaging findings. The study aimed to evaluate the effect of the timing on the efficacy of bronchoalveolar lavage in children with adenovirus pneumonia.

**Methods:**

This study included 134 patients with adenovirus pneumonia treated with BAL at Guangzhou Women and Children's Medical Center from January 2019 to January 2020.They were classified into the severe and mild groups. Based on the timing of BAL, each group was divided into the early BAL layer (received BAL within 1–9 days of the illness course) and the late BAL layer (received BAL within 10–14 days of the illness course). The clinical data of patients with different BAL timings were analyzed in two groups.

**Results:**

Among the 134 patients, 70 were categorized into the mild group and 64 were categorized into the severe group. Of the 134 patients, 42 patients received BAL early (mild group: n = 21 and severe group: n = 21) and 92 patients received BAL later (mild group: n = 49 and severe group: n = 43). In the mild group, the fever and hospital duration were shorter in patients who received BAL early than in those who received BAL later (*p* < 0.05). However, in the severe group, there were no statistically significant differences in the fever and hospital duration between patients who received BAL early and those who received BAL later. However, the need for mechanical ventilation and the incidence of BAL complications, such as new need for oxygen, were higher in patients who received BAL early than in those who received BAL later in the severe group (*p* < 0.05).

**Conclusion:**

For mild adenovirus pneumonia, early BAL may shorten the fever and hospital duration. However, early BAL in severe cases might not shorten the course of the disease or improve prognosis and may even increase the risks of mechanical ventilation and BAL complications.

## Background

Human adenovirus (HAdV) plays an important role in the development of respiratory infections in children. HAdV infections are more common in young children, mostly occurring in the first 5 years of life, with a peak incidence during the first 2 years of life [1]. Approximately 4–10% pneumonia cases are caused by HAdV [2, 3]. HAdV infections are usually mild and self-limiting in immunocompetent hosts, but some studies have shown that HAdV has the highest correlation with severe pneumonia in children, accounting for 20–33.3% severe pneumonia cases [4–6]. HAdV infection is the leading cause of death in children with severe pneumonia, with a fatality rate of up to 12% and the risk of developing long-term respiratory complications, including post-infectious bronchiolitis (PIBO), bronchiectasis, and hyperlucent lung, of up to 30% [7, 8].

HAdV can cause pneumonia and lead to high hospitalization rates in children. However, There is no specific antiviral treatment for adenoviral pneumonia. Further, intravenous immunoglobulin and systemic corticosteroids are effectively used in treating severe adenovirus pneumonia [5]. Regarding radiological manifestations, bilateral lung infiltrates or consolidation are frequently reported, and multiple lobar consolidation and pleural effusion are more common in severe cases than in mild cases [9]. Flexible fiberoptic bronchoscopy (FOB) with bronchoscopic alveolar lavage (BAL) has been widely used for the treatment of pneumonia with pulmonary consolidation or atelectasis because it effectively removes inflammatory secretions from the airway, relieves the obstruction, and reduces the damage of inflammatory reactions [10]. However, there is no standardization in the timing of BAL in children with adenovirus pneumonia.

In this study, we retrospectively analyzed 134 pediatric patients with adenovirus pneumonia who received FOB with BAL during the acute phase and evaluated the timing and safety of BAL.

## Methods

### Case definition and identification

This study enrolled 134 patients with adenovirus pneumonia who were admitted to Guangzhou Women and Children’s Medical Center between January 2019 and January 2020. The inclusion criteria were as follows:

1. Diagnosis of pneumonia according to the evidence-based guidelines published by the World Health Organization (WHO) [11].
2. Evidence of HAdV infection based on HAdV positivity on multiplex polymerase chain reaction (PCR) performed using nasopharyngeal swab, sputum, and bronchial alveolar lavage fluid samples
3. Presence of indications for BAL (e.g., radiologically proven large pulmonary lesions, lung consolidation, and atelectasis)

The exclusion criteria were as follows:

1. Chronic conditions (e.g., congenital heart disease and chronic lung disease), malignancy, severe organ dysfunction, severe protein malnutrition, confirmed or suspected active tuberculosis, immunodeficiency, and use of immunosuppressive medications before admission
2. Intolerance to BAL
3. Disagreement by parents or guardians
4. Incomplete information

Grading of the severity of the pneumonia was performed according to the guidelines of the American Thoracic Society for the management of community-acquired pneumonia. The criteria for severe pneumonia were as follows which had ≥ 1 major or ≥ 2 minor criteria [12]:

1. Major criteria: invasive mechanical ventilation, fluid refractory shock, acute need for noninvasive positive pressure ventilation, and hypoxemia requiring fraction of inspired oxygen (FiO_2_) greater than the inspired concentration or flow feasible in the general care area.
2. Minor criteria: respiratory rate greater than that recommended by the WHO classification for the age; apnea; increased work of breathing (e.g., retractions, dyspnea, nasal flaring, and grunting); PaO_2_/FiO_2_ ratio < 250; multilobar infiltrates; PEWS score > 6; altered mental status; hypotension; presence of effusion; comorbid conditions; and unexplained metabolic acidosis.

All patients were categorized into two groups based on the disease severity—mild group and severe group. Data on clinical information, laboratory results, radiological findings, and prognosis of all patients were collected.

### Study design

This retrospective observational study was conducted in accordance with the Strengthening the Reporting of Observational Studies in Epidemiology guidelines. The time, measured in days since the onset of fever (temperature, ≥ 37.5 °C) was the initial symptom of adenovirus pneumonia. Based on the onset of fever, patients were classified into the early BAL layer group (received BAL within 1–9 days of the illness course) and the late BAL layer group (received BAL within 10–14 days of the illness course).

The indicators used in evaluating the effect of different timings of BAL were as follows: (1) major indicator: recovery from illness (e.g., hospital and fever duration); (2) minor indicators: the need for advanced life support procedures (such as mechanical ventilation, continuous blood purification [CBP] and extracorporeal membrane oxygenation [ECMO]), development of pulmonary sequelae (PIBO and bronchiectasis), and outcomes (mortality). Further, we compared the incidence of adverse events, such as epistaxis, airway mucosa bleeding, laryngeal edema, adverse drug reactions, transient fever, new need for oxygen, stridor, pneumothorax, and intubation, between the two BAL layer groups [13].

### BAL under flexible bronchoscopy

Bronchoscopy and BAL for treatment were performed immediately after admission if there were no contraindications. Patients were prepared for bronchoscopy using inhalant lidocaine to minimize cough reflex, intravenous midazolam for moderate sedation, and atropine for reduction of airway secretions. BAL was performed in the most affected area, identified radiologically and/or endoscopically. The lavage includes introduction of 3–5 aliquots of sterile saline solution warmed to 37 °C, followed by immediate aspiration. The saline was recovered by aspiration into a suction trap under negative pressure of 6.65–13.3 kPa (50–100 mmHg). The recovery volume of the BAL fluid was > 40%, which is acceptable.

### Statistical analysis

Statistical analyses were performed using GraphPad Prism 8.0 (GraphPad Software Inc., San Diego, CA, USA). Data on continuous variables were skewed, and medians with ranges were used to express the summarized data. The nonparametric Mann–Whitney test was used for the two-group analysis of continuous variables. Categorical variables were assessed using Fisher’s exact test. Statistical significance was set at *p* < 0.05.

## Results

### Patients’ baseline characteristics

**Fig. 1:**
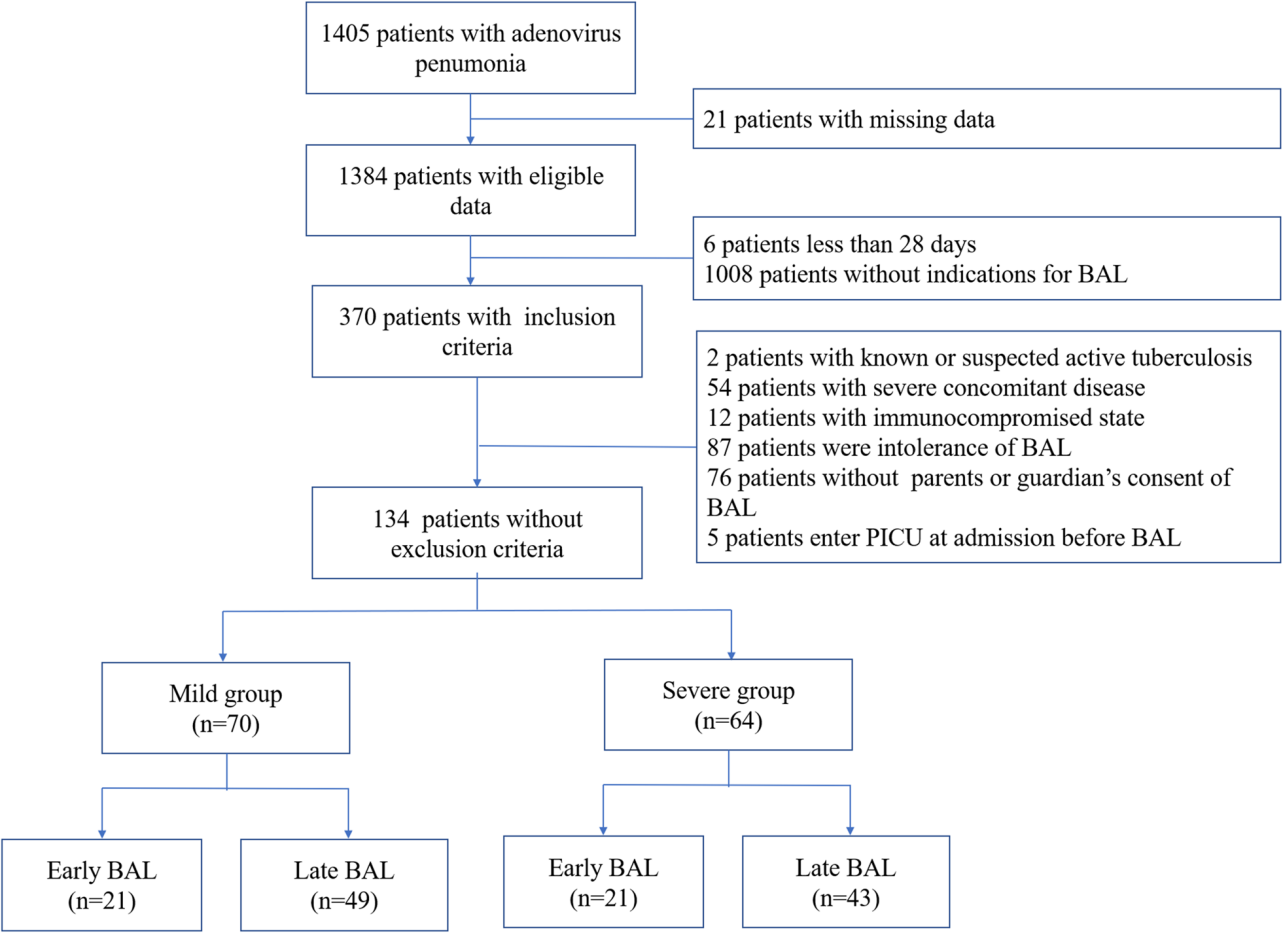
Study flowchart

**Fig. 2:**
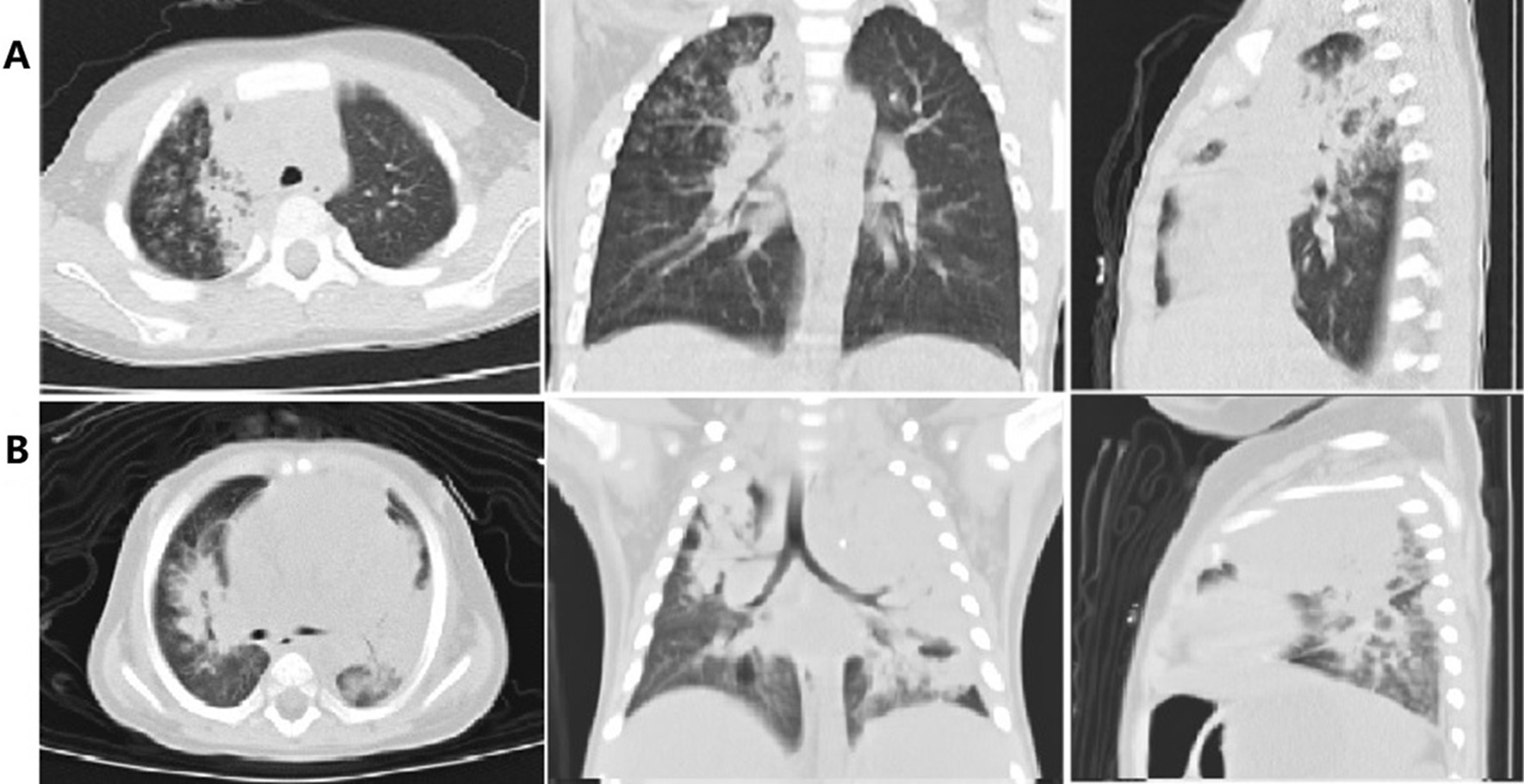
Imaging features of patients with mild and severe adenovirus pneumonia. **a** High-resolution computed tomography (CT) of the chest on the day of admission revealing diffuse infiltration and few areas of consolidation in the right upper in a child with mild adenovirus pneumonia. **b** High-resolution CT of the chest on the day of admission revealing areas of airspace consolidation in the left lobes and lingual lobe in a child with severe adenovirus pneumonia

A total of 134 patients (79 males and 55 females) were included in this study (Fig. 1), with a median age of 36 months (range, 2–144 months). Of these patients, 70 patients were diagnosed with mild adenovirus pneumonia, and 64 patients were diagnosed with severe pneumonia. Compared to patients in the mild group, those in the severe group were younger and had a longer duration of high fever. In addition, tachypnea, digestive symptoms, and changes in the level of consciousness were more frequent in the severe group than in the mild group (*p* < 0.05). The severe group had a higher probability of developing anemia and having high levels of lactate dehydrogenase (LDH) than the mild group (*p* < 0.05). Moreover, regarding radiological findings, patients in the mild group tend to have diffuse infiltrates or few areas of consolidation, while patients in the severe group tended to have multilobar consolidation and pleural effusion (Fig. 2 and Table 1).

**Table 1:** Characteristics of mild and severe adenovirus pneumonia patients on admission

Variable	Total (n = 134)	Mild group (n = 70)	Severe group (n = 64)	*P* value
*Demographic*				
Age (months), median(range)	36 (2–144)	42 (2–132)	32 (3–144)	**0.0022**
Gender, male (%)	79 (58.96)	41 (58.57)	38 (59.38)	> 0.9999
*Signs and symptoms*				
Fever, no. (%)	134 (100.00)	70 (100.00)	64 (100.00)	> 0.9999
Cough, no. (%)	124 (92.54)	64 (91.43)	60 (93.75)	0.7469
Moist crackles, no. (%)	96 (71.64)	52 (74.29)	44 (68.75)	0.5658
Tachypnea, no. (%)	45 (33.58)	0 (0.00)	45 (70.31)	** < 0.0001**
Digestive symptoms, No. (%)	21 (15.67)	4 (5.71)	17 (26.56)	**0.0015**
Change in consciousnessa, no. (%)	9 (6.72)	0 (0.00)	9 (14.06)	**0.0009**
*Laboratory findings*				
WBC(× 10^9^/L) (5–12), median (range)	7.1 (1.3–25.1)	7.1 (2.9–18.3)	7.2 (1.3–25.1)	0.9248
HGB (g/L) (105–145), median(range)	113 (56–145)	115.5 (64–136)	105.5 (56–145)	**0.0008**
HsCRP (mg/L) (< 5), median (range)	21.46 (0.3–250.3)	19.61 (0.5–182.9)	25.09 (0.3–250.3)	0.8568
LDH (U/L) (159–322), median (range)	470 (186–3440)	404 (186–980)	552 (217–3440)	** < 0.0001**
*Pulmonary imaging manifestations*				
Consolidation, no. (%)	84(62.69)	31 (44.29)	53(82.81)	**0.0004**
Pleural effusion, no. (%)	32 (23.88)	6 (8.57)	26 (40.63)	** < 0.0001**
Pneumothorax, no. (%)	1 (0.75)	0 (0.00)	1 (1.56)	0.4776

WBC: White blood cells; HGB: Hemoglobin; HsCRP: High-sensitivity C-reactive protein; LDH: Lactate dehydrogenase

The bold means *p* < 0.05, which had statistical significance

### Clinical features in different layer according to the timing of BAL

Of the 70 patients in the mild group, 21 received BAL early and 49 patients received BAL later. Of the 64 patients in the severe group, 21 patients received BAL early and 43 patients received BAL later. There was no significant difference between patients who received BAL early and those who received BAL later in the mild and severe groups in terms of demographic characteristics, respiratory manifestations (such as cough, moist crackles, and tachypnea), laboratory findings, and the proportion of severe manifestations on pulmonary imaging (such as consolidation and pleural effusion) (*p* > 0.05; Table 2).

**Table 2:** Clinical characteristics of children with mild and severe adenovirus pneumonia according to the timing of BAL on admission

Variable	Mild group (n = 70)			Severe group (n = 64)		
	Early BAL (n = 21)	Late BAL (n = 49)	*P* value	Early BAL (n = 21)	Late BAL (n = 43)	*P* value
*Demographic*						
Age (months), median (range)	44(4–96)	40(2–132)	0.6470	31(4–96)	32(3–144)	0.6601
Gender, male (%)	12(57.14)	29(59.18)	> 0.9999	13(61.90)	25(58.14)	> 0.9999
*Signs and symptoms*						
Fever, no. (%)	21(100.00)	49(100.00)	> 0.9999	21(100.00)	43(100.00)	> 0.9999
Cough, no. (%)	19(90.48)	45(91.84)	> 0.9999	20(95.24)	40(93.02)	> 0.9999
Moist crackles, no. (%)	15(71.43)	37(75.51)	0.7696	15(71.43)	29(67.44)	> 0.9999
Tachypnea, no. (%)	0(0.00)	0(0.00)	> 0.9999	15(71.43)	30(69.77)	> 0.9999
*Laboratory findings*						
WBC(× 10^9^/L) (5–12), median (range)	7.1(3.6–17.1)	7.0(2.9–18.3)	0.5078	7.1(1.5–25.1)	7.3(1.3–21.6)	0.4624
HGB (g/L) (105–145), median (range)	112(99–130)	117(64–136)	0.6103	105(66–145)	106(56–132)	0.3684
HsCRP (mg/L) (< 5), median(range)	18.6(0.5–167.5)	20.4(0.92–182.9)	0.6228	25.09 (2.52–235.4)	27.6(0.3–250.3)	0.0921
LDH(U/L) (159–322), median (range)	362(258–866)	425(186–980)	0.3874	570(266–2422)	674 (217–3440)	0.4033
*Pulmonary imaging manifestations*						
Consolidation, no. (%)	8(38.10)	23(46.94)	0.6026	16(76.19)	37(86.05)	0.3595
Pleural effusion, no. (%)	1(4.76)	5(10.20)	0.6607	7(33.33)	19(44.19)	0.4329
Pneumothorax, no. (%)	0 (0.00)	0 (0.00)	> 0.9999	0 (0.00)	1(2.33)	> 0.9999

WBC: White blood cells; HGB: Hemoglobin; HsCRP: High-sensitivity C-reactive protein; LDH: Lactate dehydrogenase

### Clinical responses to different timings of BAL in each group

**Fig. 3:**
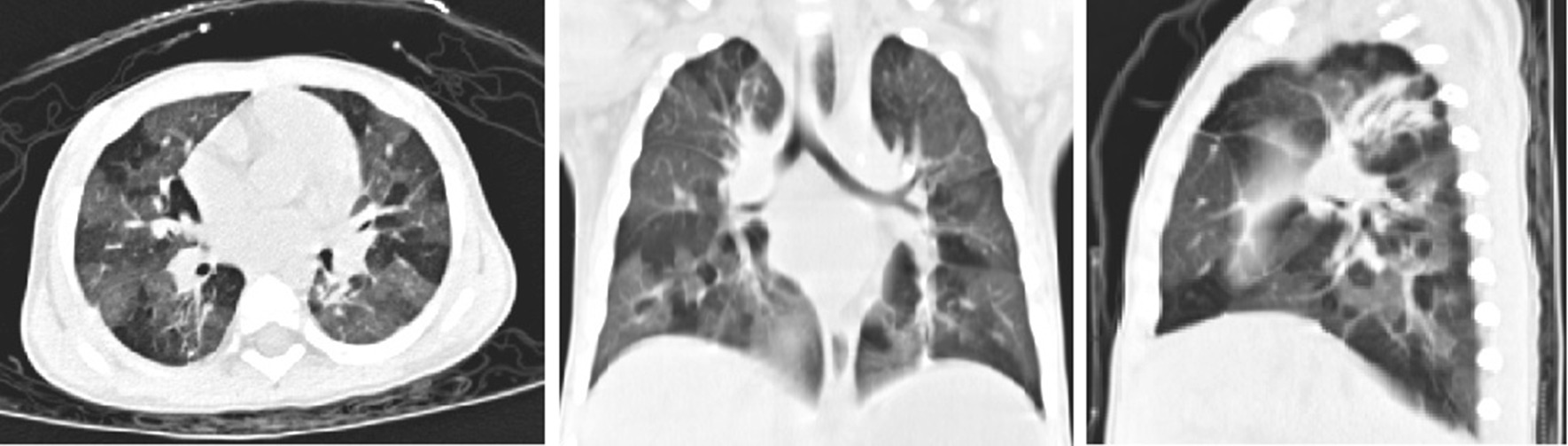
High-resolution computed tomography scan of the chest during follow-up showing bilateral mosaic ground-glass patterns with air trapping in a 22-month-old child with severe adenovirus pneumonia

The fever and hospital duration were shorter in patients who received BAL early than in those who received BAL later in the mild group (8 [5–11] days vs. 10 [5–18] days and 8 [6–12] days vs. 11 [6–19] days, respectively; *p* < 0.05 for both). However, there were no statistically significant differences in the fever and hospital duration between patients who received BAL early and those who received BAL later in the severe group (11 [7–19] days vs. 11 [7–21] days and 12 [9–29] days vs. 13 [8–34] days, respectively; *p* > 0.05 for both). Notably, patients who received BAL early required more mechanical ventilation than those who received BAL later in the severe group (*p* < 0.05). However, there was no statistically significant difference in the need for other advanced life support procedures (such as continuous blood purification and extracorporeal membrane oxygenation) between patients who received BAL early and those who received BAL later in the severe group (*p* > 0.05). In the severe group, 15 patients had left PIBO (Fig. 3), four patients had bronchiectasis, and two patients died. Furthermore, the incidence of pulmonary sequelae and mortality was not significantly different between patients who received BAL early and those who received BAL later in the severe group (*p* > 0.05; Table 3).

**Table 3:** Clinical responses of children with mild and severe adenovirus pneumonia according to the timing of BAL in the study

Variable	Mild group (n = 70)			Severe group (n = 64)		
	Early BAL (n = 21)	Late BAL (n = 49)	^*P*^ value	Early BAL (n = 21)	Late BAL (n = 43)	*P* value
*Major indicators*						
Fever duration (days), median (range)	8(5–11)	10(5–18)	**0.0287**	11(7–19)	11(7–21)	0.2670
Length of stay (days), median (range	8(6–12)	11(6–19)	**0.0006**	12(9–29)	13(8–34)	0.2893
*Minor indicators*						
*Advanced life support*						
Mechanical ventilation, No. (%)	0(0.00)	0(0.00)	> 0.9999	5(23.81)	2(4.65)	**0.0335**
CBP, No. (%)	0(0.00)	0(0.00)	> 0.9999	0(0.00)	1(2.33)	> 0.9999
ECMO, no ^.^(%)	0(0.00)	0(0.00)	> 0.9999	1(4.76)	2(4.65)	> 0.9999
*Pulmonary sequelaes*						
PIBO, No. (%)	0(0.00)	1(2.04)	> 0.9999	5(23.81)	10(23.26)	> 0.9999
Bronchiectasis, no. (%)	0(0.00)	0(0.00)	> 0.9999	1(4.76)	3(6.98)	> 0.9999
*Outcomes*						
Mortality, no. (%)	0(0.00)	0(0.00)	> 0.9999	1(4.76)	1(2.33)	> 0.9999

CBP: continuous blood purification; ECMO: extracorporeal membrane oxygenation; PIBO: post-infectious bronchiolitis

The bold means *p* < 0.05, which had statistical significance

### Security and adverse events

The incidence of common BAL complications (during and after the procedure) was not significantly different between patients who received BAL early and those who received BAL later in the mild group (*p* > 0.05). Some BAL complications, such as increased need for oxygen and intubation, were more common in patients who received BAL early than in those who received BAL later in the severe group (*p* < 0.05; Table 4).

**Table 4:** Common complications of bronchoscopic alveolar lavage

Complications	Mild group (n = 70)			Severe group (n = 64)		
	Early BAL (n = 21)	Late BAL (n = 49)	*P* value	Early BAL (n = 21)	Late BAL (n = 43)	*P* value
Epistaxis, no (%)	2(9.52)	3(6.12)	0.6324	2(9.52)	3(6.98)	> 0.9999
Airway mucosa bleeding, no. (%)	1(4.76)	2(4.08)	> 0.9999	1(4.76)	3(6.98)	> 0.9999
Laryngeal edema, No. (%)	0(0.00)	1(2.04)	> 0.9999	1(4.76)	2(4.65)	> 0.9999
Adverse drug reactions, no. %)	0(0.00)	0(0.00)	> 0.9999	0(0.00)	0(0.00)	> 0.9999
Transient fever, no. (%)	2(9.52)	2(4.08)	0.5780	2(9.52)	3(6.98)	> 0.9999
New need for oxygen, no. (%)	0(0.00)	1(2.04)	> 0.9999	7(33.33)	4(9.30)	**0.0308**
Stridor, no. (%)	0(0.00)	1(2.04)	> 0.9999	1(4.76)	2(4.65)	> 0.9999
Pneumothorax, no. (%)	0(0.00)	0(0.00)	> 0.9999	2(9.52)	0(0.00)	0.1042
Intubation, no. (%)	0(0.00)	0(0.00)	> 0.9999	5(23.81)	2(4.65)	**0.0335**

The bold means *p* < 0.05, which had statistical significance

## Discussion

HAdV plays an important role in the development of respiratory infections in children, accounting for 2–5% of all respiratory illnesses and 4%–10% of pneumonia cases [14, 15]. Although most cases are mild and indistinguishable from other viral causes, pneumonia caused by HAdV can be severe or even fatal, and it is associated with the highest risk of long-term respiratory sequelae among viral causes of pneumonia [16]. Currently, no specific therapy has demonstrated efficacy in the treatment of adenovirus pneumonia [17]. FOB with BAL has now become an important diagnostic and therapeutic procedure for respiratory diseases, including adenovirus pneumonia [18]. There is paucity of data on BAL for the treatment of adenovirus pneumonia in children. In this retrospective study of 134 subjects treated with BAL for adenovirus pneumonia at our hospital, we preliminarily assessed the potential efficacy and safety of BAL in pediatric patients with HAdV pneumonia.

The clinical manifestations of adenovirus pneumonia in children are nonspecific [2]. In our cases, children with severe adenovirus pneumonia showed serious clinical symptoms, longer fever and hospital duration. Anemia and high levels of lactate dehydrogenase were the frequently observed laboratory findings in patients with severe disease. The imaging findings in the severe cases were lung consolidation, emphysema, and pleural effusion. In particular, five patients were found to have plastic bronchitis through FOB. Therefore, FOB with BAL is an important, indispensable tool in assessing the structure and function of the lower airways and in relieving pulmonary obstructive and infiltrative lesions in pediatric patients with adenovirus pneumonia. However, there has been no research on the curative effect and the timing of BAL in children with adenovirus pneumonia.

We compared the different effects of BAL on the recovery of children with mild HAdV pneumonia (mild group) between the two layer groups (patients who received BAL within 9 days of the illness course and those who received BAL after 9 days of the illness course). The duration of fever and hospital duration was significantly shorter in patients who received BAL early than in those who received BAL later in the mild group (*p* > 0.05). All patients recovered without advanced life support, and only one patient developed pulmonary sequelae (PIBO). The incidence of operative complications of BAL was not significantly different between patients who received BAL early and those who received BAL later in the mild group (*p* > 0.05). Thus, BAL at an early stage (within 10 days of fever onset) is conducive for shortening the duration of fever and reducing the length of hospital stay without increasing operational risk.

In contrast, there was no significant difference in the duration of fever and hospital stay between patients who received BAL early and those who received BAL later in the severe group (*p* > 0.05). Furthermore, the need for advanced life support procedures (continuous blood purification and extracorporeal membrane oxygenation), the incidence of pulmonary sequelae, and mortality were not significantly different between patients who received BAL early and those who received BAL later in the severe group (*p* > 0.05). However, the need for mechanical ventilation was greater (*p* < 0.05) and some operative complications of BAL, such as increased oxygen need and intubation, were more common in patients who received BAL early than in those who received BAL later in the severe group (*p* < 0.05). There were two cases of pneumothorax among patients who received BAL early in the severe group. Therefore, early administration of BAL would not shorten the course of the disease or improve the prognosis and may increase the chance of mechanical ventilation and the occurrence of operational complications in severe cases. HAdV causing severe pneumonia can induce a more robust inflammatory cytokine storm that is quickly released at an early stage, and excessive inflammatory response can give rise to necrotizing bronchitis and diffuse alveolar damage in the bronchioles [19, 20]. Therefore, even if early BAL is performed, it is ineffective in improving the prognosis. Many children with severe adenovirus pneumonia experience rapid breathing and even hypoxia in the acute stage due to extensive lung damage. FOB with BAL itself might cause airway blockage airway trauma and tracheal spasm, resulting in operational risks such as hypoxemia or required mechanical ventilation [21]. Additionally, patients in the severe group were younger than those in the mild group. These factors result in poor tolerance to BAL and increases the risks of complications.

This study has few limitations. First, it was a retrospective study. Second, this study included a small number of subjects. Therefore, a larger prospective study is needed to confirm the role of BAL in adenovirus pneumonia. However, this study is the first to explore the clinical effect and safety of BAL in pediatric patients with adenovirus pneumonia to provide the referential data for clinical treatment.

## Conclusions

In conclusion, FOB with BAL is an important treatment for adenovirus pneumonia. Early application of BAL may provide greater benefits in mild adenovirus pneumonia. On the other hand, early BAL for severe cases may not shorten the disease course but increase the risk of complications, especially mechanical ventilation. Further large prospective studies are required.

## Data Availability

The full data and materials can be obtained from Dr. Lu (Gen Lu) upon sufficient and reasonable request.

## References

[1] Sun Q, Jiang W, Chen Z, Huang L, Wang Y, Huang F, Ji W, Zhang X, Shao X, Yan Y (2014). Epidemiology and clinical features of respiratory adenoviral infections in children. Eur J Pediatr.

[2] Shachor-Meyouhas Y, Hadash A, Kra-Oz Z, Shafran E, Szwarcwort-Cohen M, Kassis I (2019). Adenovirus respiratory infection among immunocompetent patients in a pediatric intensive care unit during 10-year period: co-morbidity is common. Isr Med Assoc J.

[3] Wu PQ, Zeng SQ, Yin GQ, Huang JJ, Xie ZW, Lu G, Jiang WH (2020). Clinical manifestations and risk factors of adenovirus respiratory infection in hospitalized children in Guangzhou, China during the 2011–2014 period. Med (Baltim).

[4] Xie L, Zhang B, Zhou J, Huang H, Zeng S, Liu Q, Xie Z, Gao H, Duan Z, Zhong L (2018). Human adenovirus load in respiratory tract secretions are predictors for disease severity in children with human adenovirus pneumonia. Virol J.

[5] Li L, Woo YY, de Bruyne JA, Nathan AM, Kee SY, Chan YF, Chiam CW, Eg KP, Thavagnanam S, Sam IC (2018). Epidemiology, clinical presentation and respiratory sequelae of adenovirus pneumonia in children in Kuala Lumpur, Malaysia. PLoS ONE.

[6] Lu MP, Ma LY, Zheng Q, Dong LL, Chen ZM (2013). Clinical characteristics of adenovirus associated lower respiratory tract infection in children. World J Pediatr.

[7] Fu Y, Tang Z, Ye Z, Mo S, Tian X, Ni K, Ren L, Liu E, Zang N (2019). Human adenovirus type 7 infection causes a more severe disease than type 3. BMC Infect Dis.

[8] Xie L, Zhang B, Xiao N, Zhang F, Zhao X, Liu Q, Xie Z, Gao H, Duan Z, Zhong L (2019). Epidemiology of human adenovirus infection in children hospitalized with lower respiratory tract infections in Hunan, China. J Med Virol.

[9] Tan D, Fu Y, Xu J, Wang Z, Cao J, Walline J, Zhu H, Yu X (2016). Severe adenovirus community-acquired pneumonia in immunocompetent adults: chest radiographic and CT findings. J Thorac Dis.

[10] Goussard P, Pohunek P, Eber E, Midulla F, Di Mattia G, Merven M, Janson JT (2021). Pediatric bronchoscopy: recent advances and clinical challenges. Exp Rev Respir Med.

[11] Li MY, Kelly J, Subhi R, Were W, Duke T (2013). Global use of the WHO pocket book of hospital care for children. Paediatr Int Child Health.

[12] Bradley JS, Byington CL, Shah SS, Alverson B, Carter ER, Harrison C, Kaplan SL, Mace SE, McCracken GH, Moore MR, St Peter SD, Stockwell JA, Swanson JT (2011). The management of community-acquired pneumonia in infants and children older than 3 months of age: clinical practice guidelines by the Pediatric Infectious Diseases Society and the Infectious Diseases Society of America. Clin Infect Dis.

[13] Costa ADS, Scordamaglio PR, Suzuki I, Palomino ALM, Jacomelli M (2018). Indications, clinical outcomes and complications of 1949 flexible bronchoscopies. Einstein.

[14] Alharbi S, Van Caeseele P, Consunji-Araneta R, Zoubeidi T, Fanella S, Souid AK, Alsuwaidi AR (2012). Epidemiology of severe pediatric adenovirus lower respiratory tract infections in Manitoba, Canada, 1991–2005. BMC Infect Dis.

[15] Jain S, Williams DJ, Arnold SR, Ampofo K, Bramley AM, Reed C, Stockmann C, Anderson EJ, Grijalva CG, Self WH (2015). Community-acquired pneumonia requiring hospitalization among U.S. children. N Engl J Med.

[16] Edmond K, Scott S, Korczak V, Ward C, Sanderson C, Theodoratou E, Clark A, Griffiths U, Rudan I, Campbell H (2012). Long term sequelae from childhood pneumonia; systematic review and meta-analysis. PLoS ONE.

[17] Lynch JP, Kajon AE (2016). Adenovirus: Epidemiology, global spread of novel serotypes, and advances in treatment and prevention. Semin Respir Crit Care Med.

[18] Hamouda S, Oueslati A, Belhadj I, Khalsi F, Tinsa F, Boussetta K (2016). Flexible bronchoscopy contribution in the approach of diagnosis and treatment of children's respiratory diseases: the experience of a unique pediatric unit in Tunisia. Afr Health Sci.

[19] Cook J, Radke J. Mechanisms of pathogenesis of emerging adenoviruses. *F1000Res.* 2017;6:90. 10.12688/f1000research.10152.1. 10.12688/f1000research.10152.1 PMC5289147 28184296

[20] Chen RF, Lee CY (2014). Adenoviruses types, cell receptors and local innate cytokines in adenovirus infection. Int Rev Immunol.

[21] Ergan B, Nava S (2018). The use of bronchoscopy in critically ill patients: considerations and complications. Exp Rev Respir Med.

